# Arousal dysregulation and executive dysfunction in attention deficit hyperactivity disorder (ADHD)

**DOI:** 10.3389/fpsyt.2023.1336040

**Published:** 2024-01-17

**Authors:** Valeria Isaac, Vladimir Lopez, Maria Josefina Escobar

**Affiliations:** ^1^Center for Social and Cognitive Neuroscience, School of Psychology, Universidad Adolfo Ibáñez, Santiago, Chile; ^2^School of Psychology, Pontificia Universidad Católica de Chile, Santiago, Chile

**Keywords:** attention deficit hyperactivity disorder, arousal dysregulation, executive dysfunction, autonomic function, state regulation

## Abstract

Attention deficit/hyperactivity disorder (ADHD) is a heterogeneous neurodevelopmental condition, that continues to have an elusive etiological background. A number of extant models and theories have historically intended to explain the many factors contributing to ADHD behaviors. One of the most accepted hypotheses has been the executive dysfunction theory associating reduction in executive control to abnormalities in structure and operational dysfunction of dopaminergic signaling networks. Nevertheless, executive functions are not always impaired in ADHD, and the literature describes other symptoms commonly reported suggesting individuals with ADHD would appear to suffer from a more general deficit. Another existing line of research, that has gained much attention recently, establishes that ADHD would have dysregulated states of brain arousal that would account for its commonly observed cognitive deficits and behavioral symptoms, described as the state regulation theory, which has now included measures of autonomic function. This article describes some important aspects that compose and challenge these two most influential theoretical constructs, executive dysfunction and state-regulation, based on their empirical evidence, implying the need to reevaluate the norms used to classify individuals and establish ADHD diagnosis. Large number of controversial results continue to exist within the study of ADHD biological and/or performance markers, possibly due to such heterogeneity and variability within the same diagnosis. The need to resolve these issues and establish newly revised diagnostic criteria for ADHD is critical, as therapeutic success depends on having accurately identified underlying neurophysiological factors in order to appropriately address them in treatment.

## 1 Introduction

Attention deficit/hyperactivity disorder (ADHD) is a neurodevelopmental disorder characterized by the presence of inattention and/or hyperactivity and impulsiveness (1) associated to disturbances in the maturation process of executive function (EF) (2, 3) and with high risk levels of developing co-morbidity with other mental disorders (such as anxiety, depression, and personality disorders). The prevalence of ADHD worldwide is approximately 3 to 9.5% (4) predominantly affecting children although many adults also suffer from personal and social impairment due to ADHD. Since it was first described as a Hyperkinetic Reaction of Childhood in the DSM-II in 1968, and later introduced as the current term of ADHD in the DSM-III-R (5), many theoretical constructs have emerged with the intention of explaining the underlying causes of this disorder. However, this diagnosis continues to be based exclusively on behavioral symptoms since many aspects of its etiology are still poorly understood (6).

One of the most currently accepted hypotheses of its underlying physiopathology is the deficit in dopamine-signaling mechanisms, associated to genetic factors encoding for dopamine receptor DRD4 and dopamine transporter DAT1, affecting prefrontal cortex, basal ganglia, thalamus, and amygdala circuits, which participate in EF (7). These neural constructs are directly relevant to the executive dysfunctional theory, introduced by Barkley (2), which suggest a reduction of executive control associated to abnormalities in frontoparietal and frontostriatal network function (8). Anatomical and functional studies have found evidence of structural differences and altered activation of the prefrontal cortex, frontoparietal and frontostriatal circuits in children with ADHD (9) supporting this hypothesis, in addition to dopaminergic and noradrenergic neurotransmitter dysfunction (10) which is critical to the operational efficiency of these circuits. This would explain impulsivity and distractibility as deficits in EF (such as in response inhibition) which would directly impact the ability to sustain attention to goal directed tasks and self-regulate social–emotional behaviors. However, the hyperactivity aspect of ADHD is largely ignored by this approach (11) in conjunction of other manifestations commonly documented in ADHD such as motor impairments (12, 13) particularly balance and gait disorders (14, 15). Despite the fact that for many decades the hypothesis that a primary EF deficit underlies ADHD behavioral symptoms has predominated in research studies, empirical findings have challenged these assumptions questioning EF deficits as a single etiology in ADHD (16–18) suggesting that poor neuropsychological performance might also relate to motivational or activation deficits (11).

A different approach explaining ADHD etiopathogeneses was developed through the state-regulation theory (i.e., cognitive-energetic model) introduced by Sanders (19) addressing neurophysiological autonomic dysregulation directly impacting brain arousal as a key underlying factor to ADHD behavioral and cognitive symptoms (20).

Arousal (or brain arousal) refers to a physiological dimension of functional brain activation comprising different levels of wakefulness in adaptation for situational requirements (21). The physiological state of arousal influences cognitive activity through locus coeruleus (LC) connectivity (22) and norepinephrine pathways, coordinated by the central autonomic network (CAN) (23). The CAN consists of interconnected areas between cortical, including insular and medial prefrontal cortices, and subcortical structures such as amygdala, hypothalamus, periaqueductal gray, parabrachial regions of pons, nucleus of the solitary tract, and ventrolateral medulla (24). The main function of the CAN is to maintain homeostasis in the current and predicted behavioral context (25) and is also integrated with affective, motivational, and cognitive processes reflected in brain function (24) which include arousal regulation (26). Optimal levels of brain arousal that induce a physiologically activated state are required to support cognitive processes (22, 24). This notion extends the original Yerkes-Dodson principle of arousal and psychophysiological engagement (27), which states the need to increase level of brain arousal in order to meet cognitive challenges. Therefore, relevant for executive functioning is the regulation of arousal through autonomic function, and the adaption of a physiological activation state (i.e., alertness, wakefulness, vigilance) to meet environmental and situational needs, so that the optimal balance between energy mobilization and conservation for responsiveness can be obtained (28).

At present state-regulation hypotheses have gained much attention and interest in research, as increasing evidence is showing altered patterns of autonomic balance in ADHD subjects, particularly in the component of arousal regulation (29) where difficulties in acquiring and sustaining an optimal physiological activation state would explain cognitive, as well as motor, performance deficits in ADHD subjects (30, 31). Arousal regulation theories of ADHD not only explain inattention and impulsivity through non-optimal regulatory processes that affect cortical EF, but also interpret hyperactivity and sensation seeking as an autoregulatory reaction to an unstable regulation of brain arousal (32) thereby addressing all 3 core aspects of ADHD diagnostic criteria.

Nonetheless, studies continue to show mixed results and large variability of how and when symptoms manifest. Many attempts have been made to identify subgroups within ADHD based on neurobiological and/or neuropsychological dimensions. Is ADHD’s disorganized behavior a consequence of deficient higher order EF control or is cognitive impairment a consequence of underlying dysregulated neurophysiological mechanisms? Another thought is the probability that ADHD diagnosis might be harboring a series of different etiologies, all sharing similar behavioral symptoms but different underlying neuropathology. If this were the case, would the study of autonomic neurophysiological indicators subserve as a means to identify a separate subgroup within ADHD? This would become relevant for more successful therapeutic treatment.

Presently, an etiological basis for ADHD continues to remain elusive, as have been efforts to subtype ADHD using biological indicators rather than solely relying on clinical assessments (18) as ADHD is considered a heterogenous condition with regard to its underlying biology and physiology, leading to variable characterizations of its symptoms. Here we will discuss part of the evidence incorporated in the theoretical constructs of executive dysfunction and state regulation hypotheses, to further suggest an integrated perspective for the observed variability in behavioral and cognitive symptoms in ADHD.

## 2 Executive dysfunction theory

EFs are defined as cognitive processes that underlie goal-directed behavior (33) involving high-order prefrontal and parietal cortical areas (34) representing a top-down approach that affects the voluntary guidance of attention, allowing for voluntary processing of relevant over irrelevant input according to task goals (35). Research on executive functions has emerged mainly on studying patients with frontal lobe damage where neuropsychological studies demonstrate severe problems in the regulation of goal-oriented behavior (36), particularly in inhibitory control, suggesting similar etiology between ADHD symptoms and presumed cognitive deficits and those of patients with frontal lobe disorders (16). The Executive Dysfunction theory suggests that the symptoms of ADHD arise wholly as a result of a reduction in executive control, caused by abnormalities in the structure, function and biochemical operation of the frontoparietal and frontostriatal neural networks (11).

In the neuropsychological assessment of ADHD, cognitive tasks are frequently used to determine whether differences in a particular neurocognitive domain would distinguish individuals with ADHD from typically developing peers. In a meta-analysis done by Frazier et al. (37), which examined the magnitude of differences in performance on several neuropsychological measures between ADHD and controls, reported that overall cognitive abilities are significantly lower among ADHD subjects, including significant impairment on all EF tasks when compared to typically developing controls. A subsequent meta-analysis by Willcutt et al. (38) looked at studies which utilized measure specifically for EF in response inhibition, working memory, set shifting, and interference control; and although results showed differences between ADHD and controls on all EF tasks suggesting that ADHD is associated with weaknesses in several key EF domains, not all aspects of EF were equally impaired, indicating that these results would not support the hypothesis that EF deficits are the single necessary and sufficient cause of all ADHD. Spatial working memory and inhibitory control, considered to be two core EF domains impaired in ADHD, failed to find group differences in a study by Brocki et al. (39) leading to conclude that these deficits are not dependent on variation in cognitive modality but rather on the effect of difficulty level or cognitive load put on the executive control system.

Deficits on non-executive functioning cognitive measures and in other developmental areas have also been reported in ADHD (40–42). For example, past studies have revealed that a subset of children with ADHD have abnormal gait and balance (43, 44) evidencing an association of these impairments with vestibular (45) and/or cerebellar dysfunction (46, 47). These findings reflect the possibility of altered underlying processes affecting cognition, but not impairment on cognition itself, calling into question the specificity of neuropsychological deficits, whether ADHD is characterized by mild global cognitive inefficiencies or by specific deficits affecting overall cognitive performance.

Brain imaging techniques have been largely applied to the study of ADHD, where magnetic resonance imaging studies have revealed smaller global brain volumes in ADHD subjects, particularly children (48, 49). Moreover, neuroimaging literature on ADHD is inconclusive with small effect sizes unable to inform clinical practice (50) as these differences are not specific to ADHD, where neuroimaging studies on other developmental disorders, such as autism, have also shown similar differences in brain volume (51, 52) when compared to controls.

From a pharmaceutical perspective, methylphenidate hydrochloride (MPH) is recommended as first line medication in clinical treatment guidelines for ADHD. The exact mechanism of action of MPH in ADHD is not completely understood, but they are presumed to act through the dopaminergic and adrenergic pathways of the frontostriatal areas in the brain by blocking the reuptake of dopamine into the presynaptic cleft (53) supported by executive dysfunction theoretical constructs on dopamine signaling deficits. The efficacy of MPH as documented in terms of reduction of at least one core symptom as determined by a parent or teacher using rating scales has been estimated at 70% (54), however, with adverse effects largely associated (most common are anorexia, insomnia, irritability) (55) and an estimated 30% of ADHD children resulting with severe negative outcomes (56) suggesting a different neural mechanism for their symptoms.

Another important aspect is that the current DSM-5 considers ADHD a neurodevelopmental condition (1) as symptoms exhibit normative change over time (57). Studies by Shaw et al. show ADHD follow a similar sequential pattern of typical cortical development yet delayed by as much as 2–3 years, depending upon the specific cortical region, concluding that the congruent delay in both cortical thickness and surface area in ADHD represents a global perturbation in the mechanisms that guide cortical maturation (58, 59). Therefore, higher order networks might not be directly impaired in ADHD, but influenced by other circuits which might be altering the optimal development and performance of higher order cognitive functions such as EF.

## 3 State-regulation theory

State-regulation theory suggests that a decreased ability to regulate arousal may contribute to the higher-level cognitive deficits in ADHD. This theory constructed in the cognitive-energetic model emphasizes a deficit of energetic (alerting) factors among ADHD patients, leading to both executive dysfunction and hyperactivity symptoms (20, 60). It states that overall efficiency of cognitive information processing is determined by state factors, also considered “energetic pools” (effort, arousal, and activation), as much as it is by computational factors (cognitive processing, executive control). The effort pool is characterized by assimilating the necessary energy to meet demands of a task, said to be activated when the current energetic state of the organism does not meet the state required to perform a task. It includes the arousal factor, defined as a phasic response that is time-locked to stimulus processing and is typically influenced by signal intensity and novelty, and behaviorally indexed by sleep–wake patterns; and the activation factor defined as the tonic physiological readiness to respond. The cognitive-energetic approach suggests that aspects of higher order executive control are dependent upon the energetic state of the individual, therefore inhibition deficits associated with ADHD may, at least in part, be due to energetic dysfunction since reduction in energy predicts failures of inhibition (61).

This follows closely the line of research earlier developed by Posner & Petersen (62) who identified three specialized attentional neural networks subtending three different attentional functions: (1) alerting or sustained attentional system– defined as achieving (phasic alerting) and maintaining (tonic alerting or vigilance) a general state of activation (or arousal) of the cognitive system for prolonged periods of time, (2) orienting or directed attentional system– defined as allocating the attentional focus to potentially relevant sensory events, and (3) executive control or selective attentional system – defined as the ability to control and inhibit impulsive responses focusing to what is relevant to the task at hand as to achieve intended goals. This attentional network perspective proposes that attention is an organic system that comprises a variety of neural processes including cortical top-down control as much as bottom-up influences (63).

The alerting system, described as the ability to prepare and sustain a vigilant state to cognitively process high priority signals as proposed by Posner and Petersen (62), has been associated to the noradrenergic system, including the right frontal and parietal lobe and the LC modulating attention and arousal (64) which allows for prolonged sustained attention to task. In other words, maintaining vigilance or sustained attention to task requires an increased and regulated arousal, with the specific role of the noradrenergic nucleus LC in the induction and regulation of cortical activation (65), attentional shifting (66) and modulating forebrain networks mediating cognitive activity, especially those related to the prefrontal cortex (22). This relationship is such that arousal level acts as a parameter which will alter the overall efficiency of cognitive performance (67). Stronger arousal reactions have been associated with better focused attention (68) emphasizing the role of heightened sympathetic activity in more efficient attention related cognitive processing (69). A number of studies have demonstrated that cognitive challenges tend to elicit increases in sympathetic activation (70) as arousal must increase to meet the energy expenditure required to invest in cognitive capacity (68). The implication of the LC in behavioral and cognitive processes probably involves a complex and dynamic interaction of LC with both subcortical structures controlling autonomic arousal and cortical structures directly involved in attentional and executive functions (22). This has led to suggest that alterations in the ability to regulate arousal may contribute to the cognitive and attentional deficits commonly found in ADHD.

Neurophysiological studies in ADHD support the hypothesis of arousal dysregulation as an underlying feature of ADHD, measuring activity from the autonomic nervous system (ANS) (29) as links between arousal and cognition are governed by interactions between central nervous system and autonomic networks (71). As previously mentioned, one of the main brainstem regions mediating interactions between ANS and cortical areas is the LC (66). The LC, one of the main sources of norepinephrine supplying the cortex, influences a range of cognitive functions including perception, working memory, and sustained attention (22) where the prefrontal cortex, essential for top-down regulation of attention and behavior, is especially sensitive to neurochemical environment, and small changes in levels of norepinephrine and dopamine produces significant changes in its functions (72).

Autonomic function measures have been used to record arousal state and change in ADHD research during cognitive and attentional effort, as LC-norepinephrine activates sympathetic networks resulting in excitatory effects increasing heart rate (HR) (73) electrodermal activity (EDA) (74) and dilation pupil size (75, 76). These ANS peripheral indices have been recorded during resting state as well as during task performance and are known to show differences between ADHD and controls (29) more often in the direction of reduced sympathetic activation in ADHD suggesting a generalized hypo-aroused state (77–79) especially during effortful cognitive tasks. For instance, lower skin conductance level (tonic component of EDA) in ADHD children during resting state (80–83) and during cognitive tasks, such in continuous performance (84) and reaction time tasks (85) have been reported. Pupil size has been found to be reduced among off-medication children and adolescents with ADHD performing a visuo-spatial working memory task, suggesting signs of hypo-arousal in ADHD resulting in specific difficulties in allocating constant and appropriate levels of attentional resources during tasks involving executive function abilities (86). Dysregulation of cortisol morning levels in ADHD have also been reported (87) suggesting difficulties in activation of sympathetic driven arousal.

Furthermore, advances in pharmaceutical development have also targeted arousal networks for ADHD treatments, as an alternative to the classically administered methylphenidate. Atomoxetine, a drug effectively used for ADHD treatment, acts as an inhibitor highly selective of noradrenergic reuptake (72) and evidences a decrease in distractibility, motor hyperactivity, and an improvement of cognitive functions contributing to the therapeutic effects of stimulants in patients with ADHD (88, 89) through stimulation of the LC-norepinephrine system. However, this is not always effective with a reported success rate of approximately 40% (90).

## 4 Integrating the evidence

At present no single model has been developed explaining all heterogeneity in ADHD symptoms. Executive dysfunction theory intends to explain ADHD disruptive and disorganized behavior from a top-down deficiency in inhibitory control but is highly dependent on task and contextual characteristics leaving several aspects of ADHD unexplained. State-regulation theory better explains variability in ADHD performance considering the activation-state and energy distribution as having direct influence on cognitive outcomes, however, during cognitive effortful tasks an optimal activation state not only must be reached but also kept at optimal level according to task demands, which would require top-down regulatory influence. This self-regulatory control not only is defined as the ability to inhibit but also to activate responses, through top-down neurocognitive processes in the service of goal attainment (91) and deliberate use of EF (92). This again calls to consider the executive dysfunction theoretical construct, however top-down activation and sustained brain arousal required for optimal cognitive performance is dependent on bottom-up energy mobilization circuits responding accordingly. Therefore, bottom-up and top-down regulatory effects function as a continuum, successive reciprocal neural feedback loops, modulating and optimizing one another during cognitive tasks (93) which results in a regulated behavior and stable performance. One of the repeated manifestations observed in ADHD is the high prevalence of intra-individual variability and inconsistency in performance (16) which may possibly be related to activation state irregularity. Findings leading to support the idea that impaired arousal regulation contributes to higher-level cognitive deficits in ADHD, is that during cognitive performance, when tasks require less effort, children with ADHD exhibit fewer cognitive impairments, reaching similar performance level as controls (30, 85, 94). Some studies show that the event rate (the speed with which stimuli are presented) affects the energetic state and level of performance in ADHD. A slow event rate (which would induce a rather underarousal/underactivation state) exhibit poor performance in ADHD with a significant slowing in reaction times (30, 95, 96), whereas a fast event rate would tend to increase arousal therefore improve attention and performance. ADHD performance improvement has been evidenced when stimulus rate is optimal (96) and when rewards are given (97) consistent with the assumption that the underlying impairment in ADHD is associated with the ability to self-regulate arousal rather than having a constant under or over aroused physiological state.

To date the origin for ADHD impairment to self-regulate arousal remains unclear. Some insights on this matter might be achieved looking at how the condition tends to evolve over time in children diagnosed with ADHD, where two important aspects should be considered. The first one, is ADHD’s high comorbidity with many other developmental disorders and conditions, reporting that as many as 60% of children with ADHD are to experience a comorbid condition across their lifespan (98). Among the most common are oppositional defiant and conduct disorders (99) as well as anxiety (100) and mood disorders (101). The second aspect of ADHD is that it is marked as being the most heritable psychiatric disorder, estimating its heritability at 80% (102) where offspring of parents with ADHD are at significant risk for ADHD and its associated psychiatric, cognitive, and educational impairments (103). Both of these features might suggest ADHD symptoms as early indicators of emerging difficulties in neurodevelopment. In other words, what we are labeling as ADHD as a single diagnosis could be the first symptomatic evidence in a spectrum of emerging future disorders. Children that begin early in development showing signs of ADHD symptoms which if not addressed in a timely manner will progressively develop into other psychiatric diagnoses later in life (for example, elevated body temperature is an early indicator of an emerging disease among many possible outcomes, but not a diagnosis in itself). Within the ADHD diagnosed group of children, a variety of underlying etiopathogeneses might be emerging. Identifying biological markers, such as autonomic manifestations, closely correlated with behavioral outcomes under specific conditions, can be a means of identifying a subgroup (possibly still at a subthreshold of becoming a psychiatric condition in the future) and tailor treatments according to more specific deficits before they develop into other diagnoses.

## 5 Conclusion

During the last two decades authors have been continuously suggesting the need to develop an updated integrative model to explain ADHD, considering the high probability that only a subset of behaviorally defined children will have deficits in a given neurocognitive mechanism believed to contribute to the disorder (17, 18, 104). To date the large amount of heterogeneity in ADHD diagnosed individuals remains and studies continue to report inconsistent results when trying to find a single underlying etiology or biological marker. Although numerous studies seem to support the hypothesis that the core problem in ADHD is an unstable arousal regulation (32, 94, 105) these continue to show mixed results (29).

The large variability and heterogeneity in performance found in children and older individuals diagnosed with ADHD supports the need for a better understanding and definition of these observed behaviors to better correlate each symptom with corresponding underlying neurobiological processes. Research is now needed to distinguish those strongest indicators and conditions for measurement that will better detect each symptom and differentiate between subjects within the ADHD diagnosis in a consistent manner, to further define subgroups or discuss an altogether differentiated diagnostic group based on neurophysiological markers. A more granular classification of a better described disorder based on known neurobiological circuits and accurate measures of these functions, would result in greater diagnostic accuracy and, most importantly, in better targeted intervention programs.

## Data availability statement

The original contributions presented in the study are included in the article/supplementary material, further inquiries can be directed to the corresponding author.

## Author contributions

VI: Conceptualization, Writing – original draft, Writing – review & editing. VL: Writing – review & editing. ME: Supervision, Writing – review & editing.
